# Myocyte-specific enhancer factor 2c triggers transdifferentiation of adipose tissue-derived stromal cells into spontaneously beating cardiomyocyte-like cells

**DOI:** 10.1038/s41598-020-80848-3

**Published:** 2021-01-15

**Authors:** Shinichiro Takashima, Soichiro Usui, Oto Inoue, Chiaki Goten, Kosei Yamaguchi, Yusuke Takeda, Shihe Cui, Yoshio Sakai, Kenshi Hayashi, Kenji Sakata, Masa-aki Kawashiri, Masayuki Takamura

**Affiliations:** 1 Department of Cardiovascular Medicine, Graduate School of Medical Science, Kanazawa University, 13-1 Takara-machi, Kanazawa, Ishikawa 920-8641 Japan; 2 Department of Gastroenterology, Kanazawa University Hospital, 13-1 Takara-machi, Kanazawa, Ishikawa 920-8641 Japan

**Keywords:** Adult stem cells, Mesenchymal stem cells, Reprogramming, Stem-cell differentiation

## Abstract

Cardiomyocyte regeneration is limited in adults. The adipose tissue-derived stromal vascular fraction (Ad-SVF) contains pluripotent stem cells that rarely transdifferentiate into spontaneously beating cardiomyocyte-like cells (beating CMs). However, the characteristics of beating CMs and the factors that regulate the differentiation of Ad-SVF toward the cardiac lineage are unknown. We developed a simple culture protocol under which the adult murine inguinal Ad-SVF reproducibly transdifferentiates into beating CMs without induction. The beating CMs showed the striated ventricular phenotype of cardiomyocytes and synchronised oscillation of the intracellular calcium concentration among cells on day 28 of Ad-SVF primary culture. We also identified beating CM-fated progenitors (CFPs) and performed single-cell transcriptome analysis of these CFPs. Among 491 transcription factors that were differentially expressed (≥ 1.75-fold) in CFPs and the beating CMs, myocyte-specific enhancer 2c (Mef2c) was key. Transduction of Ad-SVF cells with Mef2c using a lentiviral vector yielded CFPs and beating CMs with ~ tenfold higher cardiac troponin T expression, which was abolished by silencing of Mef2c. Thus, we identified the master gene required for transdifferentiation of Ad-SVF into beating CMs. These findings will facilitate the development of novel cardiac regeneration therapies based on gene-modified, cardiac lineage-directed Ad-SVF cells.

## Introduction

The morbidity burden of heart failure is growing as a result of the worldwide increase in the incidence of ischemic heart disease. Severe heart failure is resistant to medical treatment; heart transplantation is the therapy of last resort. Stem cell therapy has emerged as an adjuvant treatment for heart failure. Mesenchymal stromal cells (MSCs) and induced pluripotent stem (iPS) cells have been evaluated in preclinical and clinical trials^1–3^. iPS-cell-derived cardiomyocytes show promise for clinical applications^4,5^; however, they have several drawbacks, including tumourigenicity^6^, immunogenicity^7,8^ and immense cost. Use of MSCs presents no ethical problems and autologous MSCs have been injected into ischemic hearts^2^. The mechanism of action by which MSCs improve cardiac function is considered to be primarily paracrine^9,10^; *i.e.* secretion of growth factors and cytokines, whereas transdifferentiation into cardiomyocytes is quite limited. Additionally, the efficacy of MSCs in improving cardiac function is inadequate^11–13^. Therefore, a novel therapy based on MSCs with augmented ability to transdifferentiate into cardiomyocytes is needed.

Cardiomyocytes are terminally differentiated and their ability to promote cardiac regeneration is insufficient to maintain cardiac function when massive cardiomyocyte loss occurs following myocardial infarction and heart failure^14^. As clinical trials have shown, somatic stem cells, including bone-marrow-derived stromal cells (BMSCs) and adipose tissue-derived stromal vascular fraction (Ad-SVF), have advantages in terms of safety and availability over iPS cells^2,15^, but they rarely differentiate into spontaneously beating cardiomyocytes. Several groups have achieved transdifferentiation of bone-marrow or Ad-SVF into spontaneously beating cardiomyocyte-like cells (CMs); however, supplementation with DNA methyltransferase 1 inhibitor, growth factors, or cytokines, or use of a semi-solid medium, was required^16–18^. Additionally, because a small number of CMs was produced, their characteristics could not be evaluated in detail. Here, we developed an experimental protocol under which murine SVF cells from subcutaneous adipose tissue reproductively transdifferentiated into spontaneously beating CMs without induction. We also identified the beating CM-fated progenitors (CFPs) from which the beating CMs originated. By evaluating the global transcriptome of CFPs and of beating CMs, we investigated the mechanism underlying the transdifferentiation of SVF cells into CMs.

## Results

### Production of beating SVF cell clusters in SVF primary culture

**Figure 1:**
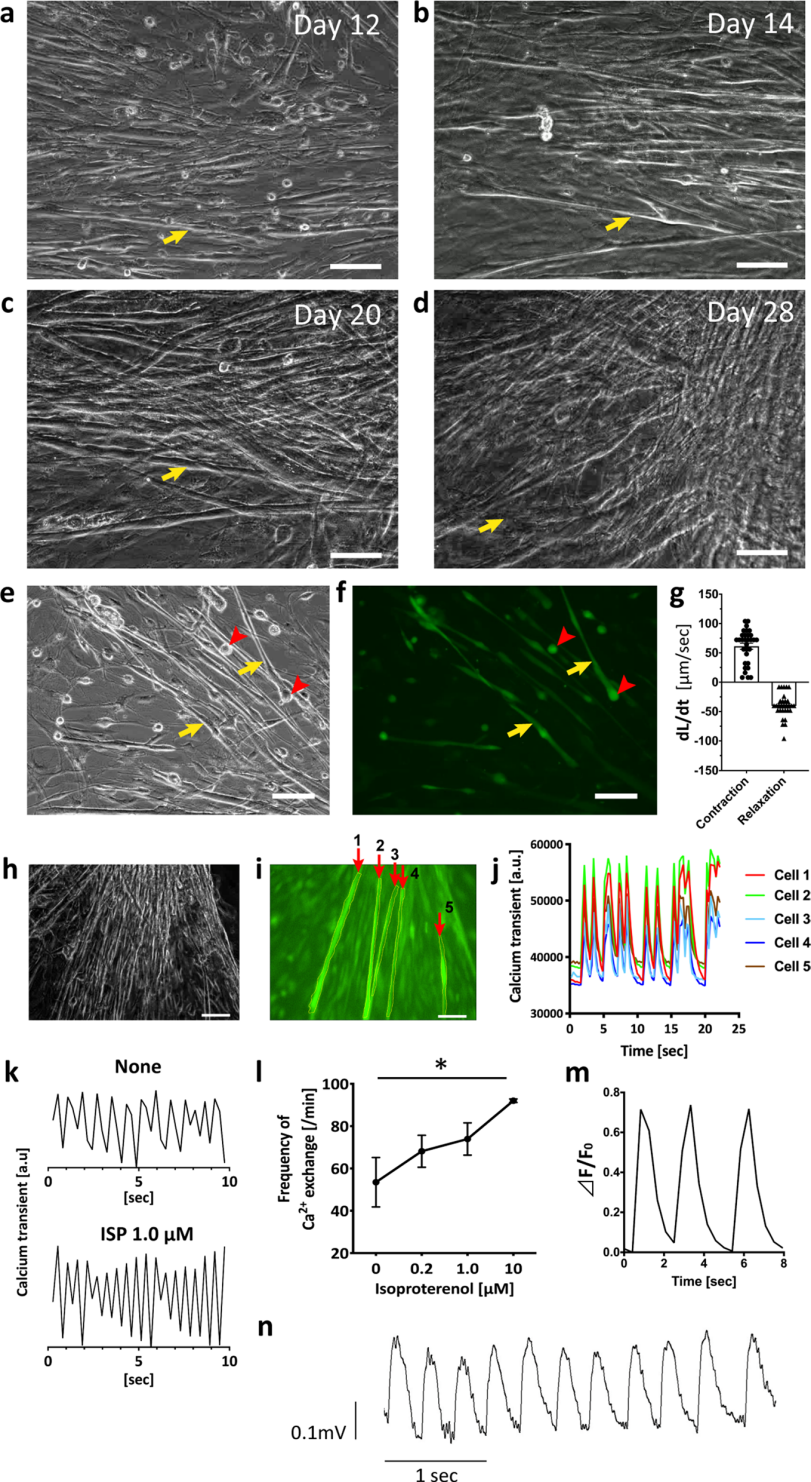
SVF partially transdifferentiates into spontaneously beating cells without induction. (**a**–**d**) Representative phase-contrast images of a growing colony of beating cells on days 12 (**a**), 14 (**b**), 20 (**c**), and 28 (**d**) in a primary culture of SVF extracted from the inguinal subcutaneous fat pad. The arrows show identical cells. (**e**,**f**) Phase-contrast (**e**) and corresponding calcium ion fluorescence image of SVF beating clusters loaded with the calcium indicator fluo-8/AM (**f**) on day 14. The arrowhead indicates a round cell with intracellular calcium ion oscillation. Arrow shows an elongated cell that is changing from the round form and beginning to beat spontaneously. (**g**) Maximal rate of shortening (dL/dt) and relaxing (–dL/dt) in each cycle of contractions of a representative isolated SVF beating cell on day 28. (**h**–**j**) Synchronised contraction of beating SVF cells on day 28. Phase-contrast image of a cluster of beating SVF cells (**e**) and the corresponding calcium ion fluorescence image (**i**). Transient calcium waves of the five cells (**j**) traced in yellow in panel I (arrows 1 to 5). (**k**) Transient calcium wave of beating SVF cells with or without ISP. (**l**) The frequency of calcium ion exchange in beating SVF cells in response to ISP. ISP; isoproterenol. Scale bar, 100 µm. * *p* < 0.05; *** *p* < 0.001 by *t*-test or ANOVA. (**m**) A representative calcium ion transient trace, represented as ΔF/F_0_, for a single beating SVF cell. (**n**) A representative action potential of a spontaneously beating SVF that was recorded on day 28 of primary culture. The action potential duration to 50% repolarisation (APD_50_) was 180 ± 3 and that to 90% repolarisation (APD_90_) was 277 ± 15 ms.

SVF cells isolated from the inguinal subcutaneous fat pad of adult mice were induced on standard uncoated polystyrene tissue culture plates with minimal growth medium (GM) (see Methods) without any other induction. From 12 to 14 days of primary culture, cells of various morphologies were evident: a cluster of preadipocytes, fibroblast-like cells (data not shown), and several clusters of round cells among spindle-shaped cells (Fig. 1a). The round cells changed into an elongated-tube-like form and began to beat spontaneously (Fig. 1b,c). After 20 to 28 days, the beating cell colony became larger as the number of cells increased, forming branches and connecting to adjacent cells, and forming a cluster of beating cells (Fig. 1d and Supplementary Videos S1–S6). These beating cell clusters continued beating for a few months with regular changes of medium (data not shown). The mean number of beating SVF cell colonies on day 28 was 9.2 ± 0.7 per 100 mm dish, accounting for ~ 0.15% of all cells, whereas no beating cells were observed among passage 3 adipose tissue-derived stem cells (ASCs) (Supplementary Figure S1). The characteristics of SVF cells and ASCs are also shown in Supplementary Figure S1. The beating SVF cells demonstrated intracellular calcium oscillation, which began at the round phase and was more marked at the elongated-tube-like phase (Fig. 1e,f; Supplementary Videos S7 and S8). The contraction and relaxation were rhythmical along the long axis of the cells (Fig. 1g). As shown in Fig. 1h,j; Supplementary Video S9, the SVF cells in a beating cluster showed synchronised contractions, indicating that they were electrically connected. The beating rate of the cells differed according to the conditions, such as the number of cells in the cluster, and isoproterenol increased the beating rate in a dose-dependent manner (Fig. 1k,l), showing that the contractions were mediated by adrenergic beta-receptor. In a beating SVF cell, the trace of the intracellular calcium ion transient demonstrated that the radical increase in fluorescence was followed by a gradual decrease to baseline (Fig. 1m). The action potential of a beating SVF cell on day 28 of primary culture was characterised by an initial rapid depolarisation phase that merged with a terminal repolarisation phase with a small plateau phase (Fig. 1n). The mean action potential durations to 50% (APD_50_) and 90% (APD_90_) of repolarisation were 180 ± 3 and 277 ± 15 ms, respectively. We also examined whether the efficiency of producing beating SVF cells using minimal GM was comparable with that reported using GM. The use of minimal GM tended to yield more beating SVF cells, compared with the complete GM, which also includes growth factors and cytokines (see Methods) (Supplementary Figure S2).

### Global transcriptome analysis of SVF-beating cells

**Figure 2:**
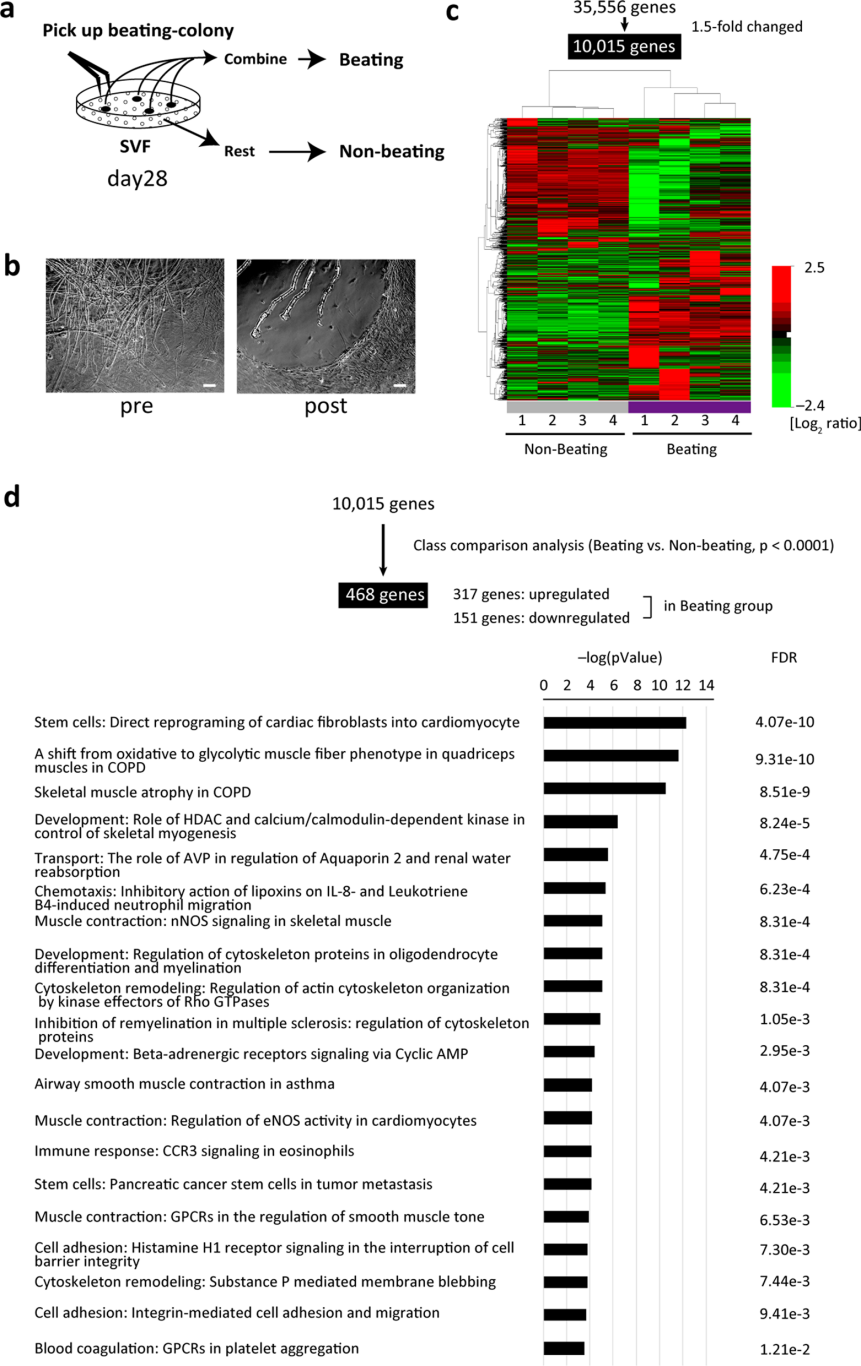
Global transcriptome analysis of SVF beating cell clusters. (**a**) Schematic of RNA extraction from beating (beat) and non-beating (non-beat) cells. (**b**) Phase-contrast images of a beating cell cluster before (pre) and after (post) being picked-up. Scale bar, 100 µm. (**c**) Heatmap image of microarray data clustered using an unsupervised learning method, showing DEGs between beating and non-beating SVF cells using 10,015 prefiltered genes (under the condition that at least one expression value is at least 1.5-fold changed in either direction from the median value; n = 4 per group). The scale extends from 0.19- to 5.7-fold over the median (–2.4 to + 2.5 on a log_2_ scale). Red indicates increased expression, whereas green indicates decreased expression. The graphical image of heatmap was generated using BRB Array Tools software (v. 4.6.0) (NIH, Bethesda, MD, USA) (URL: https://brb.nci.nih.gov/BRB-ArrayTools/). (**d**) Gene pathways altered in the beating group among 468 genes extracted by class comparison analysis (*p* < 0.0001) from 10,015 prefiltered genes (FDR: 10^−7^ to 0.002). FDR, false discovery rate.

**Figure 3:**
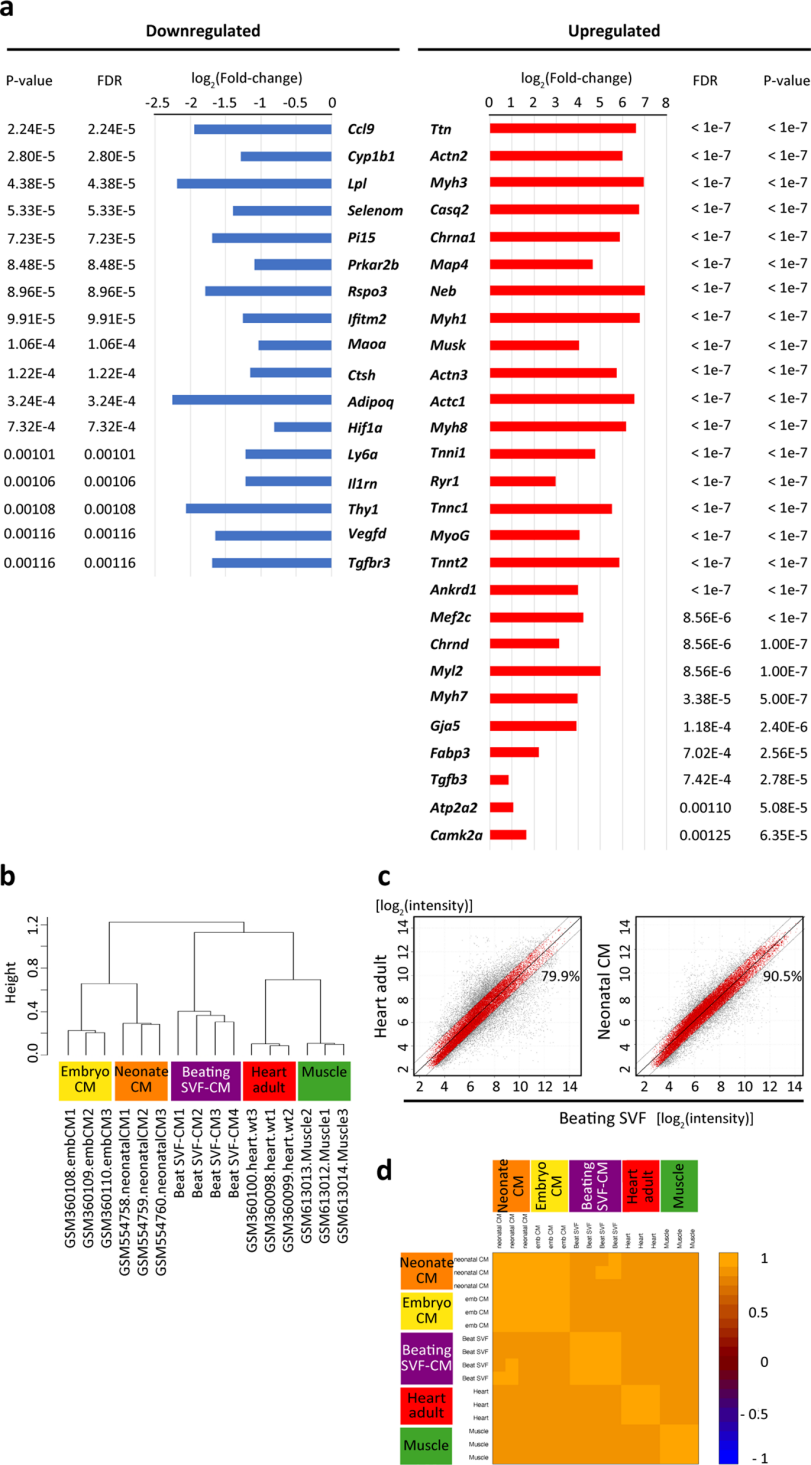
Global gene expression profile in beating clusters of SVF. (**a**) Primary genes up- and downregulated in the beating compared to the non-beating group among 468 genes extracted by class comparison analysis (*p* < 0.0001) from 10,015 prefiltered genes (FDR: 10^−7^ to 0.002). FDR, false discovery rate. (**b**) Hierarchical clustering analysis of 32,000 genes (genes with a log-ratio variation in the 10th percentile were excluded) using an unsupervised learning method, compared with the SVF group based on array (Affymetrix Mouse Gene 1.0 ST Array) data from the gene expression omnibus database (NIH; Table S2). (**c**) Scatterplot of gene expression [log_2_(intensity)] between beating SVF and adult heart or neonatal cardiomyocytes. Genes with a ≤ twofold difference in expression are shown in red and the ratios to all genes are indicated. (**d**) Image plot of pairwise Pearson correlation of expression of arrays of beating SVF comparing to neonatal cardiomyocytes, embryo cardiomyocytes, adult heart, and skeletal muscle. The arrays are ordered based on hierarchical clustering using the average linkage method. Colours indicate the correlation coefficients. The graphical images of hierarchical clustering (**b**), scatter plot (**c**), and image plot (**d**) were generated using BRB Array Tools software (v. 4.6.0) (NIH, Bethesda, MD, USA) (URL: https://brb.nci.nih.gov/BRB-ArrayTools/).

To investigate the phenotype of the beating SVF cells, we performed a global transcriptome analysis. All of the beating SVF colonies in a dish (~ 100 ng total RNA) on day 28 were manually picked up and combined, constituting the beating-SVF (beating) group. The other cells were collected as the non-beating-SVF (non-beating) group (Fig. 2a,b). Using 10,015 pre-filtered genes, the heatmap image of the microarray expression data showed that the beating group was separated from the non-beating group (Fig. 2c). The class comparison analysis extracted 317 upregulated and 151 downregulated genes in the beating group. Among these genes, pathway analysis revealed several associated with ‘direct reprogramming of cardiac fibroblasts into cardiomyocytes’ (Fig. 2d). In the beating group, genes associated with sarcomere, ion channels, and transcription factors related to cardiomyocytes as well as skeletal muscle (Fig. 3a) were upregulated, whereas stem-cell-related genes, such as Ly6a and Thy1, were downregulated. Next, we performed a hierarchical clustering analysis of 32,000 genes in adult heart and skeletal muscle tissue and cardiomyocytes isolated from murine embryo or neonatal ventricle (Supplementary Table S2). As shown in Fig. 3b, beating SVF clustered adjacent to neonatal cardiomyocytes and adult cardiac tissue. A scatterplot demonstrated that the gene expression profile of beating SVF was 90.5% similar (< 2.0-fold) to that of neonatal cardiomyocytes, and 79.9% to that of adult heart tissue (Fig. 3c). A pairwise Pearson correlation plot showed that beating SVF was highly correlated with neonatal cardiomyocytes (Fig. 3d).

### The phenotype of SVF-beating cells was similar to ventricular cardiomyocytes

**Figure 4:**
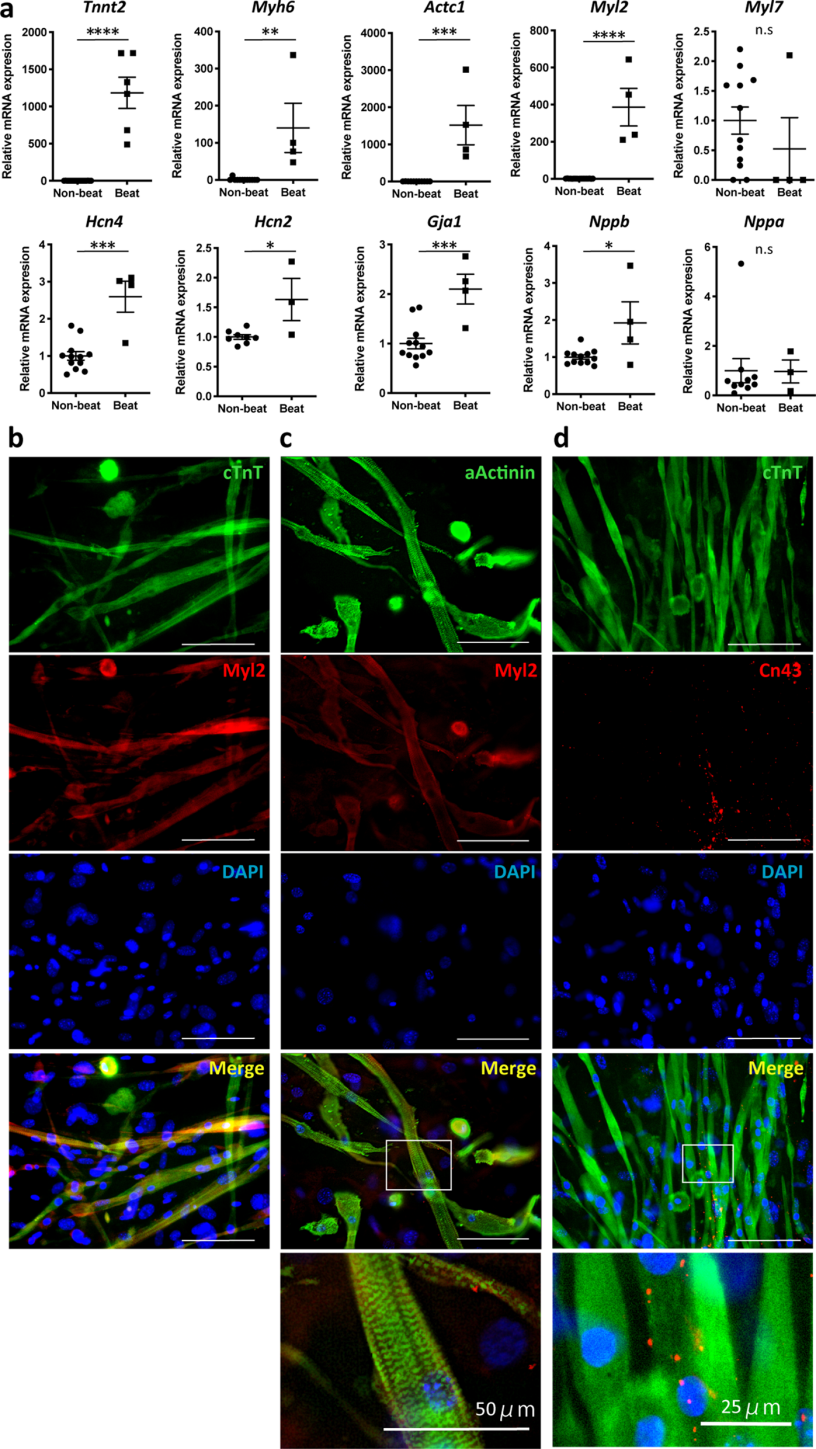
SVF beating cells have a cardiomyocyte phenotype. (**a**) mRNA levels of cardiomyocyte-specific sarcomere proteins, ion channels, and secreted proteins in the beating and non-beating groups. (**b**) Representative immunofluorescence images of beating cells stained for cardiac troponin T (cTnT), Myl2, alpha-actinin (aActinin), and connexin-43 (Cn43) on day 28 after cultivating SVF. White square, area of interest (shown below at higher magnification). Scale bar, 100 µm. * *p* < 0.05; ** *p* < 0.01; *** *p* < 0.001; **** *p* < 0.0001 by *t*-test. DAPI, 4′,6-diamidino-2-phenylindole; SVF, stromal vascular fraction.

Because the beating SVF clusters had a similar global gene expression profile to that of neonatal cardiomyocytes, we examined the expression of cardiomyocyte-related genes and the levels of the encoded proteins. qRT-PCR demonstrated that the beating SVF cells expressed genes encoding cardiomyocyte-specific sarcomere proteins, including cardiac muscle troponin T (Tnnt2), myosin heavy chain 6 (Myh6), and actin alpha cardiac muscle 1 (Actc1). The ventricular phenotype of myosin light chain (MLC) 2v (Myl2) was expressed in the beating SVF cells but the atrial type of MLC 2a (Myl7) was not expressed in the beating SVF, as in the non-beating SVF. Likewise, immunocytological analysis demonstrated that the beating SVF cells co-expressed cardiac troponin T (cTnT) with MLC2v (Fig. 4a,b). Co-staining of alpha-actinin with MLC2v indicated the beating SVF cells to be striated muscle with a ventricular phenotype (Fig. 4c). Expression of the pacemaker ion channels encoded by Hcn4 and Hcn2 was increased in beating SVF cells. Expression of the gene encoding secreted brain natriuretic peptide (BNP), but not atrial natriuretic peptide, was enhanced in beating SVF cells, consistent with the expression of ventricular-type MLC. The beating SVF expressed Gja1 at a high level and connexin 43 protein was localised between beating cells (Fig. 4a,d). In the non-beating SVF group, adipocyte- and chondrocyte-specific genes were upregulated (Supplementary Figures S3a and S3c), and some non-beating SVF cells differentiated into adipocytes in the same dish in which beating SVF cells grew (Supplementary Figure S3b).

### Analysis of round beating CFPs

**Figure 5:**
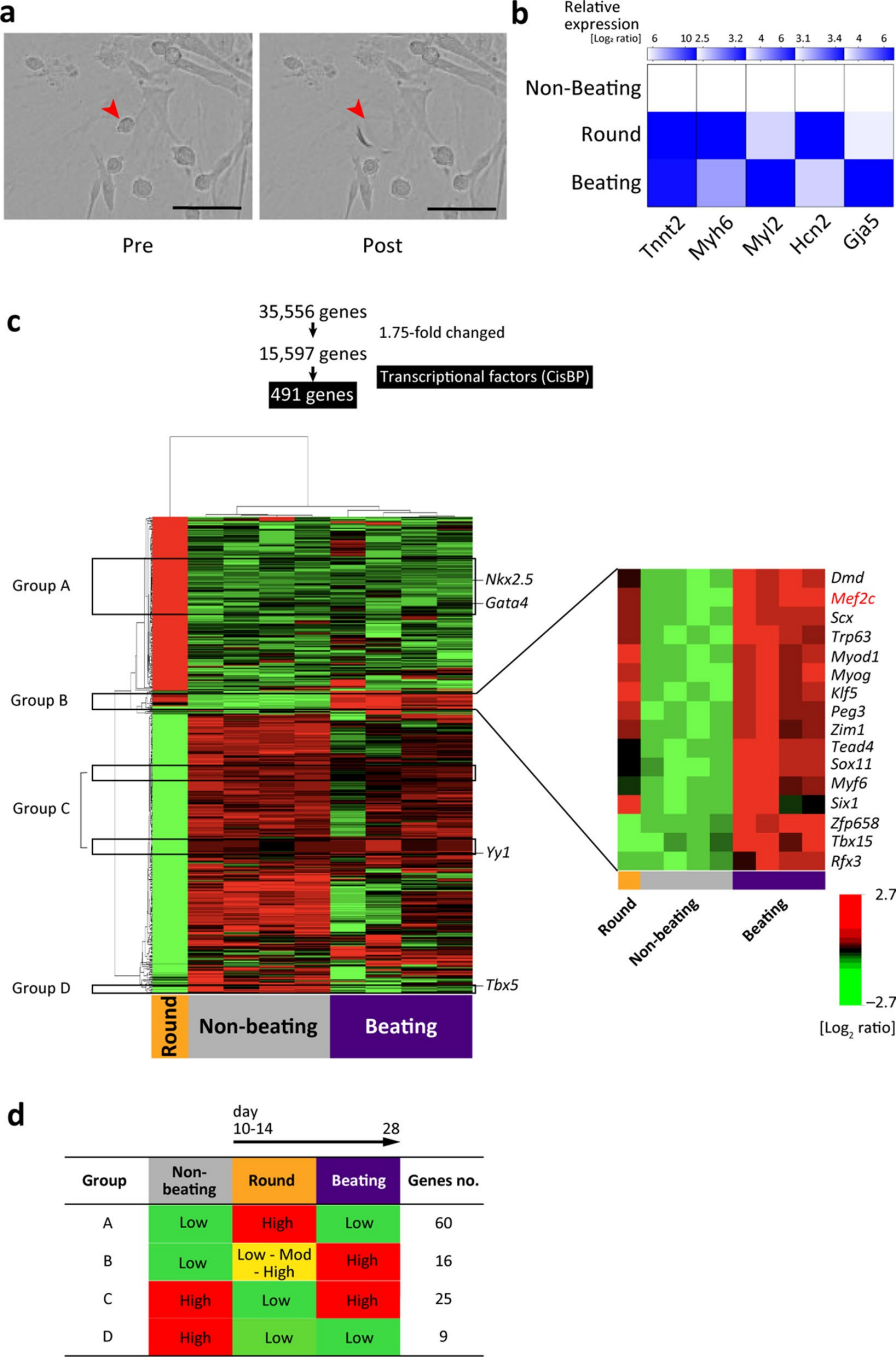
Single cell-based analysis of round cells, which appear at ~ day 14 and transdifferentiate into beating CMs. (**a**) Representative phase-contrast images of a round cell (red arrowhead) before (pre) and after (post) being picked-up using the ALS CellCelector. Scale bar = 100 µm. (**b**) Relative expression levels of representative cardiac-specific genes. Colours indicate log_2_ ratios. (**c**) Heatmap of microarray data showing 491 differentially expressed transcription factors in the round, non-beating, and beating groups using 15,597 prefiltered genes (under the condition that at least one expression value has a ≥ 1.75-fold change in either direction from the median value; n = 4 in the non-beating and beating group versus n = 1 in the round cell group. The round cell group constituted the combined product of six isolated cells). Among 491 genes, four groups of transcription factors (A to D) were selected (black boxes). Group B is magnified, and the key genes are indicated at right. The scale extends from 0.15- to 6.5-fold over the median (–2.7 to + 2.7 in log_2_ scale). Red indicates increased expression, and green indicates decreased expression. (**d**) Alterations of gene expression and numbers of genes are shown. The graphical images of expression panel (**b**) and heatmap (**c**) were generated using BRB Array Tools software (v. 4.6.0) (NIH, Bethesda, MD, USA) (URL: https://brb.nci.nih.gov/BRB-ArrayTools/).

To test our hypothesis that the round cells were the progenitors of CMs, we performed a single-cell-based global gene expression analysis. On day 14 of primary SVF culture, six round cells were picked up using the ALS CellCelector system (Fig. 5a) and combined for microarray analysis. The round cells expressed genes related to cardiac sarcomere components, gap junctions, and pacemaker activity (Fig. 5b). To investigate the key transcription factors for transdifferentiation into CMs, 491 CisBP transcription factors were identified by screening for unchanged genes among the beating, non-beating, and round cells. The heatmap classified these genes into four groups according to their expression pattern: group A, upregulated only in round cells; group B, slightly upregulated in round cells and substantially upregulated in beating cells; group C, downregulated only in round cells; and group D, downregulated in round and beating cells (Fig. 5c,d). Nkx2–5, which is important for differentiation into the cardiac lineage in the embryo, was upregulated only in round cells.

### Mef2c was upregulated in round CFPs and in beating CMs

**Figure 6:**
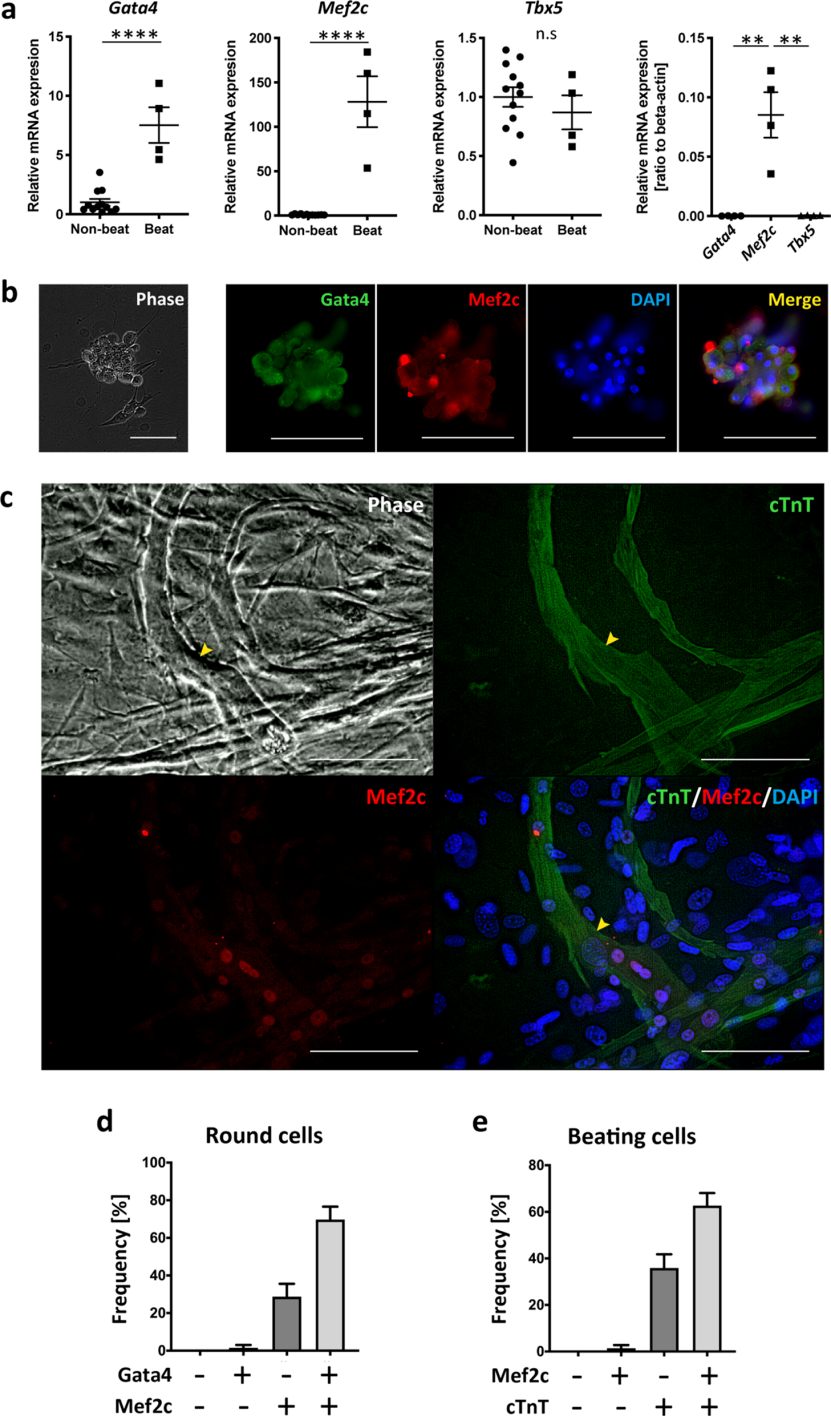
Gata4, Mef2c, and Tbx5 expression in the beating and non-beating groups. (**a**) Left three panels, gene expression levels in the beating and non-beating group. Right panel, gene expression levels in the beating and non-beating group relative to that of β-actin. (**b**) Representative immunofluorescence images of a cluster of round cells expressing Gata4 and Mef2c on day 14. (**c**) Phase-contrast and immunofluorescence images of beating cells expressing cardiac troponin T (cTnT) and Mef2c on day 28. The arrowhead indicates a beating cell. Scale bar, 100 µm. DAPI, 4′,6-diamidino-2-phenylindole, ** *p* < 0.01; **** *p* < 0.0001 by *t*-test or ANOVA and Tukey’s post hoc test. (**d**) The frequency of round cells expressing Gata4 and Mef2c (10 round cells were examined per experiment, n = 13). (**e**) The frequency of beating cells expressing Mef2c and cTnT (15 beating cells were analysed per experiment, n = 4).

Because the gene expression profile of beating SVF was suggestive of ‘direct reprogramming of cardiac fibroblasts into cardiomyocytes’ (Fig. 2d), we examined the expression of the associated transcription factors Gata4, Mef2c, and Tbx5. Gata4, Mef2c, and Tbx5 showed different expression patterns. They were assigned to groups A, B, and D as described above, respectively (Fig. 5c). qRT-PCR confirmed that Gata4 and Mef2c were upregulated in beating SVF, whereas expression of Tbx5 was unchanged, compared to that in non-beating SVF (Fig. 6a). Mef2c was markedly upregulated in beating SVF. Furthermore, immunofluorescence imaging demonstrated that Mef2c and Gata4 were coexpressed in 70% of the round cells (Fig. 6b,d). Moreover, 98% of the beating SVF cells were positive for cardiac troponin T, and 63% of those expressed Mef2c in the nucleus (Fig. 6c,e, and Supplementary Video S10). Since the pathway analysis of the global transcriptome (Fig. 2d) indicated that the beating SVF clusters expressed skeletal muscle- and cardiomyocyte-related genes, we examined the expression of MyoD1, a specific marker of skeletal myocyte differentiation. Flow cytometry demonstrated that on day 14, 9.1% of the SVF cells were positive for MyoD1, and 4.5% were positive for cardiac troponin T (Supplementary Figure S4a). Among the cardiac troponin T-positive cells on day 14, most of which were round cells as confirmed by immunocytochemistry (Supplementary Figure S4c), 81% expressed MyoD1 (Supplementary Figure S4b). The round cells no longer expressed MyoD1 upon becoming elongated beating SVF cells (Supplementary Figure S4c). On day 28, most of the beating SVF cells expressing cTnT were negative for MyoD1 (Supplementary Figures S4a, S4b, and S4d). By contrast, a non-beating SVF cluster expressing MyoD1 was negative for cTnT (Supplementary Figure S4e).

### Overexpression of SVF with Mef2c augmented the yield of round CFPs and their transdifferentiation into CMs

**Figure 7:**
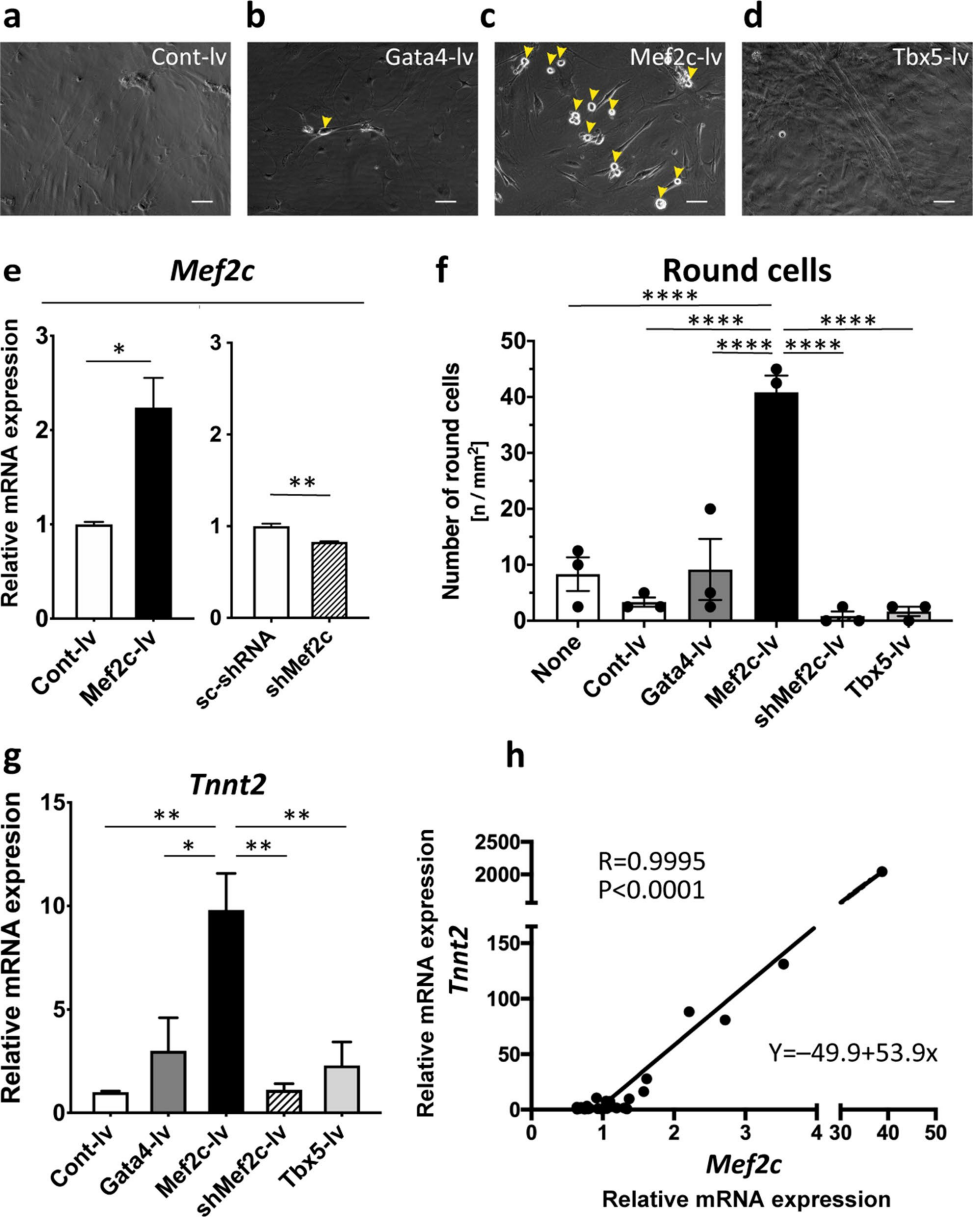
Overexpression of SVF with Mef2c augments the number of round cells and their transdifferentiation into CMs. (**a**–**d**) Phase-contrast images of SVF on day 28 after transfection on day 5 (**a**, control; **b**, Gata4; **c**, Mef2c; **d**, Tbx5). Arrowheads indicate round cells; lv, lentivirus vector. (**e**) mRNA level of Mef2c, determined by qRT-PCR, on day 28 in SVFs transfected with the control vector and Mef2c-lv (left), or scrambled shRNA and shMef2c-lv (right), on day 5, respectively. shRNA, short hairpin RNA; shMef2c, shRNA targeting Mef2c. (**f**) Number of round cells on day 14 after introducing SVF with none (none) or the control, Gata4, Mef2c, shMef2c, or Tbx5 lentivirus vector. (**g**) mRNA level of cardiac troponin T by qRT-PCR on day 28 in SVF transfected with the control, Gata4, Mef2c, shMef2c, or Tbx5. (**h**) Relationship between the mRNA levels of Mef2c and cardiac troponin T in the SVF transduced with Mef2c. R, Pearson’s product-moment correlation coefficient. The linear interpolation formula is displayed. Scale bar = 100 µm. * *p* < 0.05; ** *p* < 0.01; **** *p* < 0.0001 by ANOVA and Tukey’s post hoc test.

To examine whether direct reprogramming-related transcription factors regulate the transdifferentiation of SVF into CMs, we transduced SVF with transcription-factor-encoding genes using a lentivirus vector. Overexpression of Mef2c resulted in a 16-fold increase in the number of round cells (Fig. 7a–d,f) and significantly increased expression of cardiac troponin T (Fig. 7g). Both effects were suppressed by gene silencing using a short hairpin RNA targeting Mef2c (Figs. 7e–g). By contrast, overexpression of Gata4, Tbx5, or control vector had no effect. Although the efficiency of transducing with Mef2c varied among the experiments, expression levels of cardiac troponin T showed a positive linear correlation with those of Mef2c (Fig. 7h). SVF with 40-fold augmented Mef2c gave rise to spontaneously beating CMs with ~ 2000-fold increased cardiac troponin T expression. Therefore, Mef2c positively regulates the transdifferentiation of SVF into CMs.

## Discussion

We identified simple culture conditions under which murine inguinal Ad-SVF reproducibly and spontaneously transdifferentiated into beating CMs without induction. This phenomenon occurred only in primary SVF culture and was mediated by round cells. We evaluated the global gene expression profiles of the spontaneously differentiated beating CMs and the round progenitor cells. We also identified the master gene required for SVF transdifferentiation into beating CMs.

MSCs are multipotent mesenchymal stem cells that have advantages over iPS-derived CMs^3,6,8,19^ in terms of safety and availability^20^. The mechanism of action of MSCs in cardiac regeneration therapy is protection of injured cardiomyocytes by secreted cytokines and growth factors. Several failed attempts have resulted in a consensus that MSCs do not differentiate into beating CMs^9,21,22^. Bone-marrow-derived stromal cell lines can be induced to differentiate into spontaneously beating CMs by 5-azacytidine^16^. Subcutaneous Ad-SVF shows promise for clinical applications because the need for expansion in culture^13,15^ is removed by the abundant multipotent stem cells that can transdifferentiate into adipocytes, chondrocytes, or cardiomyocytes^23,24^. Planat-Bénard et al. reported transdifferentiation of Ad-SVF into beating CMs using semi-liquid medium and supplementation with cytokines and growth factors^17^. The use of semisolid culture with enzyme-crosslinked gelatine hydrogels obviates the need for supplements to induce differentiation of SVF into beating CMs^18^. By contrast, we achieved differentiation of SVF into spontaneously beating CMs using an uncoated culture dish and standard liquid medium supplemented only with a reducing agent. By minimising external modification of SVF cells, their original characteristics when undergoing transdifferentiation into CMs were revealed, without reducing the efficiency of beating SVF cell production. In this study, we performed immunofluorescence imaging, intracellular calcium imaging, electrophysiological recording, and single-cell-based global gene expression profiling of beating SVF clusters.

Expression profiling of beating SVF clusters was hampered by the presence of non-beating SVF. In this study, beating SVF clusters were manually selected and RNA was extracted for global gene expression profiling. Consistent with previous reports^17,18^, the beating SVF clusters expressed genes encoding cardiac sarcomere, gap junction, pacemaker ion channel, and secretory proteins. The levels of the corresponding proteins (cardiac troponin T, ventricular myosin light chain, and connexin 43) were confirmed by immunofluorescence. The global gene expression profiles of the beating SVF clusters were comparable to the profiles of neonatal cardiomyocytes^25,26^ but differed from those of adult cardiomyocytes. In an electrophysiological study, the beating CMs had a prolonged action potential with a small plateau phase. Consistently, the intracellular calcium influx decreased gradually in the systolic phase, similar to previous studies of a rodent ventricular phenotype^27^. Collectively, the beating SVF cells were similar to cardiomyocytes.

The factor that regulates the switch to the cardiomyocyte lineage during SVF primary culture is important. In adults, cardiomyocytes are terminally differentiated and do not grow^14^. Transcription of cardiomyocyte-related genes is strictly regulated by epigenetic processes including CpG methylation, histone modifications, and noncoding RNAs^28–31^, which restrict the activation of transcription factors related to cardiac differentiation^29^. A pathway analysis of the microarray data showed that the expression of genes related to ‘direct reprogramming of cardiac fibroblasts into cardiomyocytes’ was altered in beating SVF cells. Direct reprogramming shows promise for regeneration therapy^26^, in which application of cardiac fibroblasts in combination with transcription factors—including Gata4, Mef2c, and Tbx5—induces transformation into cardiomyocytes^32^. Indeed, in the beating SVF group, Gata4 was upregulated and Mef2c was highly expressed; by contrast, expression of Tbx5 was unchanged. Additionally, in the non-beating SVF group, adipocyte- and chondrocyte-specific genes were upregulated. Therefore, reprogramming of the cells occurred during the primary culture of SVF, and the reprogrammed cells differentiated into cardiomyocytes, as well as adipocytes or chondrocytes. The ultimate goal of the Ad-SVF-derived CMs is to selectively generate abundant functional CMs for cardiac regeneration applications. The addition of supplements including cytokines and growth factors, as used previously^17^, did not increase the yield of beating CMs. However, we found that the transcription factor Mef2c was crucial for activating SVF cells towards cardiac differentiation. Transduction of the SVF cells with Mef2c using a lentiviral vector allowed sustained augmentation of gene expression during cell multiplication, which increased CM generation. Notably, the number of beating CMs was directly proportional to Mef2c expression. Therefore, our results indicate that primed SVF cells transduced with Mef2c have therapeutic potential in heart failure.

The fact that the beating SVF cells originated from round cells led to the hypothesis that the latter were CFPs. The round cells were readily discriminated from other cells by phase-contrast microscopy, enabling isolation of-single cells^33^ and assessment of their global gene expression profiles. The expression of transcription factors could be classified into four groups, and some cardiomyocyte-specific genes were upregulated even in round cells. Also, Mef2c was upregulated in round cells and this was retained in beating SVF; by contrast, upregulation of Gata4 and Nkx2–5 was transient. These results imply that the round cells were CFPs. The CFPs expressed specific genes, including those of cell-differentiating antigens (data not shown), which are candidate markers of CFPs. Note, however, that the CFP transcriptome in this study was the result of one round of the experiment based on a pooled sample of six hand-picked cells, raising the concern that noise was introduced; we hope to verify this in our next study by using more samples.

Our results also raise several questions. First, the global transcriptome of the beating SVF group represented a mixed cardiomyocyte/skeletal myocyte phenotype. On day 28, when the microarray analysis was performed, immunofluorescence staining revealed that the beating SVF cells all expressed cardiac troponin T, but not MyoD1, which is a specific transcription factor regulating skeletal muscle differentiation^34^. By contrast, a non-beating cell cluster expressing MyoD1 was negative for cardiac troponin T. Therefore, we considered a beating SVF cell to be a cardiomyocyte, but not a skeletal myocyte. As we selected the beating SVF clusters manually, we speculated that they contained not only beating SVF cells but also round cells (mostly expressing MyoD1) and nearby non-beating skeletal myocytes. We postulated that was why the transcriptome of the beating SVF group represented a mixed cardiomyocyte/skeletal myocyte phenotype. Using an immunofluorescence approach, the round cells considered to be CFPs exhibited transient MyoD1 expression; subsequently, the CFPs no longer expressed MyoD1 upon becoming cTnT-positive beating SVF cells. Although MyoD1 is a master gene involved in skeletal muscle^34^, its expression was induced under hyperoxic conditions in cultured chick foetal cardiac myocytes^35^. Furthermore, fusion of the MyoD domain to Mef2c, Gata4, Hand2, and Tbx5, increased the efficiency of direct reprogramming of fibroblasts into cardiomyocytes^36^. From these observations, we speculate that the transient expression of MyoD1 in round cells plays a crucial role in their differentiation into cardiomyocytes. The mechanism underlying the regulation of differentiation of SVF cells into beating cardiomyocytes or non-beating skeletal myocytes remains unclear. However, the duration of MyoD1 expression might be a key. We will address this using a time-lapse tracing of CFPs or MyoD1-fate mapping in our next study. We also evaluated miRNA expression in the beating SVF and round CFPs (data not shown). miRNAs have the potential to modulate the differentiation of stem cells^37–39^. Thus, identification of the key microRNA that regulates the differentiation of CFPs against skeletal myocytes but toward cardiomyocytes is considered a reasonable approach in the development of “pure” cardiomyocytes^40^, which will be investigated in our next study. Second, it is of note that spontaneous cardiac differentiation occurred in SVF primary culture but not in subcultured cells. Li and co-workers reported that Gata4, Tbx5, and Baf60c induced differentiation of repeated cultured adipose-derived stromal cells into CMs, albeit on a small scale^41^. Because the phenotype of MSCs changes during subculture^42^, additional key factors other than Mef2c that are essential for spontaneous differentiation into beating CMs but are lost in the course of repeated subcultures should also be identified. Third, the significance of Tbx5 in the differentiation of SVF cells into beating CMs in this study was unclear. Tbx5 is a key transcription factor regulating cardiac differentiation and is essential for direct remodelling of fibroblasts into cardiomyocytes^26^. The role of Tbx5 in SVF cell differentiation could be evaluated by its knockdown, which we will perform in our next study. Fourth, the beating CMs had a ventricular, rather than an atrial, phenotype and expressed MLC2v and BNP. According to a previous report^43^, during embryonic development of the four-chambered heart, expression of MLC2v is regulated by Mef2c and Nkx2–5 and represses ANF expression in the ventricle. Further investigation of the regulation of differentiation of SVF into chamber-specific cardiomyocytes is needed. Finally, this was an in vitro study. In previous clinical trials, intramyocardial injection of skeletal myoblasts failed to improve heart function due to ventricular arrhythmia caused by electrical uncoupling^44^. Although the beating CMs expressed connexins 40 and 43 and demonstrated synchronised contraction, whether the beating CMs are functional in a transplanted heart in vivo needs further study. Furthermore, the administration of Mef2c-primed SVF or augmented CM-fated round cells in an animal model of heart failure is needed to investigate their safety (arrhythmogenicity) and efficacy (engraftment and improving ventricular function). In conclusion, we identified the master genes required for Ad-SVF transdifferentiation into beating CMs. The findings will enable the development of novel cardiac regeneration therapies using gene-modified, cardiac lineage-directed Ad-SVF.

## Methods

All experiments were carried out in accordance with approved guidelines and were approved by the Animal Experiment Committee at the Institute for Experimental Animals, Kanazawa University.

### Isolation of Ad-SVF

Bilateral inguinal subcutaneous adipose tissues without lymph nodes, weighing ~ 500 mg, were obtained from a C57BL/6J mouse (aged 8–12 weeks; male or female). After washing with sterile phosphate-buffered saline (PBS), the tissues were digested using the Tumor Dissociation Kit, Mouse (Miltenyi Biotec, Bergisch Gladbach, Germany) in serum-free Dulbecco’s modified Eagle’s medium (DMEM)/F-12 (Thermo Fisher Scientific, Waltham, MA, USA) for 30 min at 37 °C using gentleMACS (Miltenyi Biotec). The digested tissue was thoroughly pipetted ~ 10 times in DMEM supplemented with 10% foetal bovine serum (FBS) (F7524, Sigma-Aldrich, St. Louis, MO, USA) and filtered through a 100-µm nylon mesh, followed by centrifugation at 400× *g* for 5 min. After washing with DMEM/F-12 with FBS and centrifugation, the cell pellet was resuspended in BD Pharm Lyse (BD Biosciences, Franklin Lakes, NJ, USA) and incubated for 5 min to lyse red blood cells. After a further round of centrifugation, the cell pellet was filtered through a 35-µm mesh and centrifuged at 400× *g* for 5 min; then, the SVF was resuspended in culture medium.

### Cell culture

#### Primary culture of SVF cells

Cells in the SVF were counted using a TC20 automated cell counter (Bio-Rad Laboratories, Hercules, CA, USA) and seeded on a standard uncoated tissue culture plate (Nunc) (Thermo Fisher Scientific) in minimal GM consisting of Iscove’s Modified Dulbecco's Medium (IMDM; I3390, Sigma-Aldrich), 10% FBS, 2 mmol/L L-glutamine (Wako, Osaka, Japan), 100 units/mL penicillin, 100 µg/mL streptomycin, and 0.1 mmol/L 2-mercaptoethanol (StemSure) (Wako) at a density of 5000 live cells/cm^2^, and then cultured under standard conditions (37 °C with 5% CO_2_). For a comparative experiment, complete GM was also used, as reported previously. The complete GM consisted of minimal GM supplemented with 10 μg/mL recombinant human insulin (Sigma-Aldrich), 200 μg/mL human transferrin (Sigma-Aldrich), 10 ng/mL recombinant murine IL-3 (R&D Systems, Minneapolis, MN, USA), 10 ng/mL recombinant mouse IL-6 (R&D Systems), and 50 ng/mL recombinant mouse SCF (R&D Systems). The culture medium was changed twice weekly for 4 weeks.

#### ASCs

To obtain ASCs, the SVF cells were passaged at sub-confluence, and those from passage 3 were used as ASCs.

#### Oil red O staining

After washing with PBS, cultured cells were fixed in 4% paraformaldehyde, stained with Oil Red O working solution for 20 min, and washed three times in PBS. After rinsing with 60% isopropanol, the stained cells were observed under a microscope.

### Intracellular calcium ion imaging

The beating SVF was washed with PBS twice and loaded with 4 µM Fluo-8AM ester (AAT Bioquest, Sunnyvale, CA, USA), in Hank’s and 4-(2-hydroxyethyl)-1-piperazineethanesulfonic acid buffer (HHBS, AAT Bioquest) for 30 min at 37 °C. The cells were washed twice with HHBS and fluorescence images were obtained using a fluorescence microscope (BZ-9000, Keyence, Tokyo, Japan) operating in video-recording mode (15 frames/s). The transient calcium wave of targeted beating cells was visualised in a frame-by-frame manner using the Fiji plug-in for ImageJ software. Temporal variations in the brightness of selected areas corresponded to the transient calcium level of the selected cells. The rhythmic wave of the cell was plotted based on the time interval of the video frames. The change in fluorescence intensity was reported as ΔF/F_0_, where ΔF = Fr − F_0_, Fr is the recorded (absolute) intensity, and F_0_ is the average baseline intensity of the cell during quiescence.

### Action potential recordings

The action potential of the beating SVF cells was recorded on day 28, as described previously^45^. Briefly, the action potential was recorded using patch pipettes at 37℃ with an external control solution containing (in mmol/L) NaCl 140, KCl 5.4, CaCl_2_ 1.8, MaCl_2_ 2, glucose 10, and HEPES 10 (pH 7.4). Suction pipettes were generated from borosilicate capillary tubes (8250 glass; A-M Systems) and fire-polished to obtain a resistance of 5–8 MΩ when filled with a solution containing (in mmol/L) KCl 120, EGTA 5, K_2_ATP 5, MgCl_2_ 2.5, and HEPES (pH 7.2). Under the Axiovert 10 (Carl Zeiss, Jena, Germany) microscope, Vm was measured using the AxoClamp 2A amplifier (Molecular Devices) in bridge mode via the disrupted patch technique, as described previously^43^. Vm was digitised using the PowerLab system (AD Instruments, Dunedin, New Zealand) and recorded at a sampling frequency of 2 kHz/16-bit in LabChart 7.0 (AD Instruments) using a 0.5–40 Hz band-pass filter. All recording equipment was housed on an air table within a grounded Faraday cage to minimise background noise. The APD_50_ and APD_90_ of repolarisation were calculated from a series of 10 potentials.

### Assessment of contraction and relaxation

Raw phase-contrast video files of spontaneously beating cells were imported into Kinovea v. 0.8.15. A small region of interest (ROI) on the beating CMs was defined as the tracking point. Acceleration in each frame at the point of interest was extracted using the software in semi-automatic mode, wherein a tracking point is placed and the software attempts to automatically track those pixels into the next frame. The maximum rates of contraction and relaxation for each cycle were determined by extracting the acceleration data in a frame-by-frame manner.

### Pharmacological study

A fluorescence video of beating CMs of interest was obtained through intracellular calcium imaging, and a calcium transient wave was observed. Next, the frequency of calcium ion exchange was calculated. The chronotropic response was assessed by adding 0.2 to 10 µM isoproterenol (Sigma-Aldrich) to CMs.

### Extraction of RNA from beating cell clusters

Beating cell clusters were manually picked from a 100-mm dish using tweezers (MA50, Dumond, Switzerland) under an inverted optical microscope (CKX41, Olympus, Tokyo, Japan) at 40–100 × magnification, and then transferred to rinsing buffer (Buffer RA1 added to Tris (2-carboxyethyl) phosphine [TCEP]; NucleoSpin RNA XS [Merchery-Nagel, Düren, Germany]). The beating cell colonies obtained from two or three dishes derived from one lot of SVF were combined into one sample, which was processed for RNA extraction without addition of carrier RNA, according to the manufacturer’s instructions. Other cells in the dish were collected as non-beating cells and subjected to RNA extraction. Total RNA was extracted from unselected cells using the ISOSPIN Cell & Tissue RNA Kit (Nippon Gene, Tokyo, Japan). The quality of the RNA was verified (RNA integrity number > 8) using a bioanalyser (Agilent 6000 Nano Assay, Agilent Technologies, Santa Clara, CA, USA).

### Picking single round cells

Single round cells were picked using an ALS CellCelector (ALS, Jena, Germany). The CellCelector combines an automated high-content imaging cytometry platform with automated cell micromanipulation using a vertical high-precision glass micro-capillary fixed to a precision robotic arm and a syringe pump. Briefly, scanning of round cells of interest in a phase-contrast channel was followed by automated identification of the target cells and recovery into a destination vessel (PCR tube) for downstream analysis.

### Microarray analysis

#### Preparation of RNA and microarray hybridisation

For microarray analysis, the quality of RNA was confirmed (RNA integrity number > 8) using a bioanalyser (RNA 6000 Nano Assay, Agilent Technologies). Total RNA (50 ng) was processed to generate sense-strand cDNA (ss-cDNA) using the Ovation Pico RNA WTA System (Tecan Genomics, Maennedorf, Switzerland). Biotin-labelled RNA or DNA fragments were hybridised to GeneChip Mouse Gene 1.0 ST Arrays (Affymetrix, Santa Clara, CA, USA) and quantified. Images were processed and cell intensity (CEL) files were generated using GeneChip Command Console software (Affymetrix).

#### Data analysis

Raw data were imported into BRB Array Tools software (v. 4.6.0) (NIH, Bethesda, MD, USA) (URL: https://brb.nci.nih.gov/BRB-ArrayTools/) and log_2_-scaled, normalised-mean-centred, and subjected to average-linkage clustering. Hierarchical clustering of both gene expression (rows) and the independent samples (columns) in the data matrix was performed. The rows/columns of the data matrix were re-ordered such that the most similar expression patterns or samples were located adjacent to each other. To identify genes that were differentially expressed among the groups, the class comparison tool, which is based on univariate *t*-tests, of BRB-Array Tools was used. Gene Ontology analysis was performed using the functional ontology enrichment tool of MetaCore v. 19.4 (Clarivate Analytics, Tokyo, Japan). Data for genes that were differentially expressed among the groups were imported with the corresponding fold-changes and *p* values.

### Quantitative PCR

Total RNA (100 ng) was used to generate cDNA using TaqMan Universal Master Mix. Quantitative real-time polymerase chain reaction (qRT-PCR) was performed using an ABI Prism 7300 Sequence Detection System (Applied Biosystems, Foster City, CA, USA). Beta-actin (Mouse ACTB, 4352341E) was used as an endogenous control. Relative expression was assessed using the comparative CT method, correcting for the amplification efficiency of the primers, and each sample was analysed in duplicate (TaqMan assay IDs are listed in Supplementary Table S1).

### Immunocytochemistry and microscopy

For immunocytochemical analysis, the SVF was seeded on an uncoated Nunc Lab-Tek Chamber slide (Thermo Fisher Scientific) at a density of 5000 live cells/cm^2^ (20,000 live cells/well). The cells were fixed in 4% paraformaldehyde at room temperature (RT) for 15 min. After permeabilisation and blocking with 0.5% Triton X-100 in PBS and 1% bovine serum albumin (BSA) in PBS, the cells were incubated with the following primary antibodies at the indicated dilutions: mouse anti-cardiac troponin T (1:200, ab8295, Abcam, Cambridge, UK), rabbit anti-Myl2 (1:100, 10,906–1-AP, Proteintech Group, Chicago, IL, USA), rabbit alpha-actinin (1:100, ab137346, Abcam), mouse alpha-actinin (1:800, A7811, Sigma-Aldrich), rabbit anti-connexin43 (1:500, ab11370, Abcam), mouse anti-Gata4 (1:50, sc-25310, Santa Cruz Biotechnology, Dallas, TX, USA), rabbit anti-MyoD1 (1:100, NBP1-54153SS, Novus Biologicals, LCC, Centennia, CO, USA), and rabbit anti-Mef2c (1:200, GTX105433, GeneTex, Irvine, CA, USA) overnight at 4 °C. Next, the samples were incubated with 1% BSA containing the following secondary antibodies: Alexa Fluor 488- or 594-conjugated anti-rabbit or -mouse (1:400, all from Invitrogen) IgG (H + L) at RT for 30 min, and mounted in VECTASHIELD (Vector Laboratories, Burlingame, CA, USA) containing 4′,6-diamidino-2-phenylindole (DAPI). Stained slides were observed under a fluorescence microscope.

### Flow cytometry

Cells were washed twice with PBS and trypsinised to generate cell suspensions. For cell surface antigen staining, the cells were blocked with an anti-CD16/CD32 antibody (553,141, BD PharMingen, Franklin Lakes, NJ, USA) and incubated for 15 min with the optimal dose of anti-CD90-APC (553,007, BD PharMingen), anti-CD34-FITC (560,238, BD PharMingen), anti-CD140a (562,777, BD PharMingen), or isotype control antibody. For intracellular antigen staining, the cells were permeabilised and fixed using BD Cytofix/Cytoperm for 20 min before incubating another 20 min with an optimal dose of anti-cardiac troponin T-FITC (130–106-745, Miltenyi Biotec) and anti-mouse MyoD1(NB100-56511SS, Novus Biologicals, LCC) antibodies conjugated to APC using the LYNX Rapid APC Antibody Conjugation Kit (LNK034APC, Bio-Rad Laboratories). The cells were analysed using a BD Accuri C6 flow cytometer (BD Biosciences). Data analysis was performed using FlowJo ver. 10 software (BD Biosciences).

### Transduction of SVF

SVF was transduced with Mouse LentiORF Particles (pLenti-C-mGFP-P2A-Puro; Origene, Rockville, MD, USA): Mef2c (NM_025282, MR226865L4V), Gata4 (NM_008092, MR227022L4V), or Tbx5 (NM_011537, MR227369L4V) for overexpression, or LentiORF Control Particles (pLenti-C-mGFP-P2A-Puro, PS100093V, Origene) as an empty vector control. For silencing of mouse Mef2c, LentiORF lentiviral particles of mouse Mef2c-specific short hairpin RNA (TL511428V, Origene) or scrambled control were used. Cells at ~ 50% confluence on days 5 to 7 after seeding of SVF at 5000/cm^2^ on 12-well culture plates were incubated for 24 h in 500 µL of minimal medium at a multiplicity of infection of 10. Subsequently, the lentivirus-containing medium was replaced with fresh medium plus puromycin (5 µg/mL, A11138-03, Gibco) to select for successfully transduced cells. Puromycin-resistant colonies were observed under a microscope.

### Statistics

Data are reported as mean ± standard error. Comparisons of groups were undertaken using Student’s *t*-test or analysis of variance (ANOVA) followed by Tukey’s post hoc test, as appropriate, in GraphPad Prism 8 (San Diego, CA, USA).

## Supplementary Information

Supplementary Information 1.

Supplementary Information 2.

Supplementary Information 3.

Supplementary Information 4.

Supplementary Information 5.

Supplementary Information 6.

Supplementary Information 7.

Supplementary Information 8.

Supplementary Information 9.

Supplementary Information 10.

Supplementary Information 11.

## Data Availability

The microarray data have been deposited at the National Center for Biotechnology Information Gene Expression Omnibus (GEO) under accession code: GSE154301.
