# Supplementary material for: Myocyte-specific enhancer factor 2c triggers transdifferentiation of adipose tissue-derived stromal cells into spontaneously beating cardiomyocyte-like cells
Source: Sci Rep. 2021 Jan 15;11:1520. doi: 10.1038/s41598-020-80848-3 (PMC7810870; doi:10.1038/s41598-020-80848-3)
Supplement: Supplementary file 1 — Supplementary Information 1. [file 41598_2020_80848_MOESM1_ESM.pdf]

Supplementary Information files

Title:

Myocyte-specific enhancer factor 2c triggers transdifferentiation of adipose tissue-derived stromal cells into spontaneously beating cardiomyocyte-like cells

Authors:

*Shinichiro Takashima[1], Soichiro Usui[1], Oto Inoue[1], Chiaki Goten[1], Kosei Yamaguchi[1], Yusuke Takeda[1], Shihe Cui[1], Yoshio Sakai[2], Kenshi Hayashi[1], Kenji Sakata[1], Masa-aki Kawashiri[1] and Masayuki Takamura[1]

Institutional affiliations:

1. Department of Cardiovascular Medicine, Graduate School of Medical Science, Kanazawa University, Kanazawa, Ishikawa, 920-8641, Japan
2. Department of Gastroenterology, Kanazawa University Hospital, Kanazawa, Ishikawa, 920-8641, Japan

Address:

1. 13-1 Takara-machi, Kanazawa, Ishikawa, 920-8641, Japan
2. 13-1 Takara-machi, Kanazawa, Ishikawa, 920-8641, Japan

Contact info

***Correspondence:** takashin@m-kanazawa.jp

Shinichiro Takashima, MD, PhD

Department of Cardiovascular Medicine, Graduate School of Medical Science, Kanazawa University

13-1 Takara-machi, Kanazawa, Ishikawa, 920-8641, Japan

Tel: +81-76-265-2259

Fax: +81-76-265-4210

Email: takashin@m-kanazawa.jp

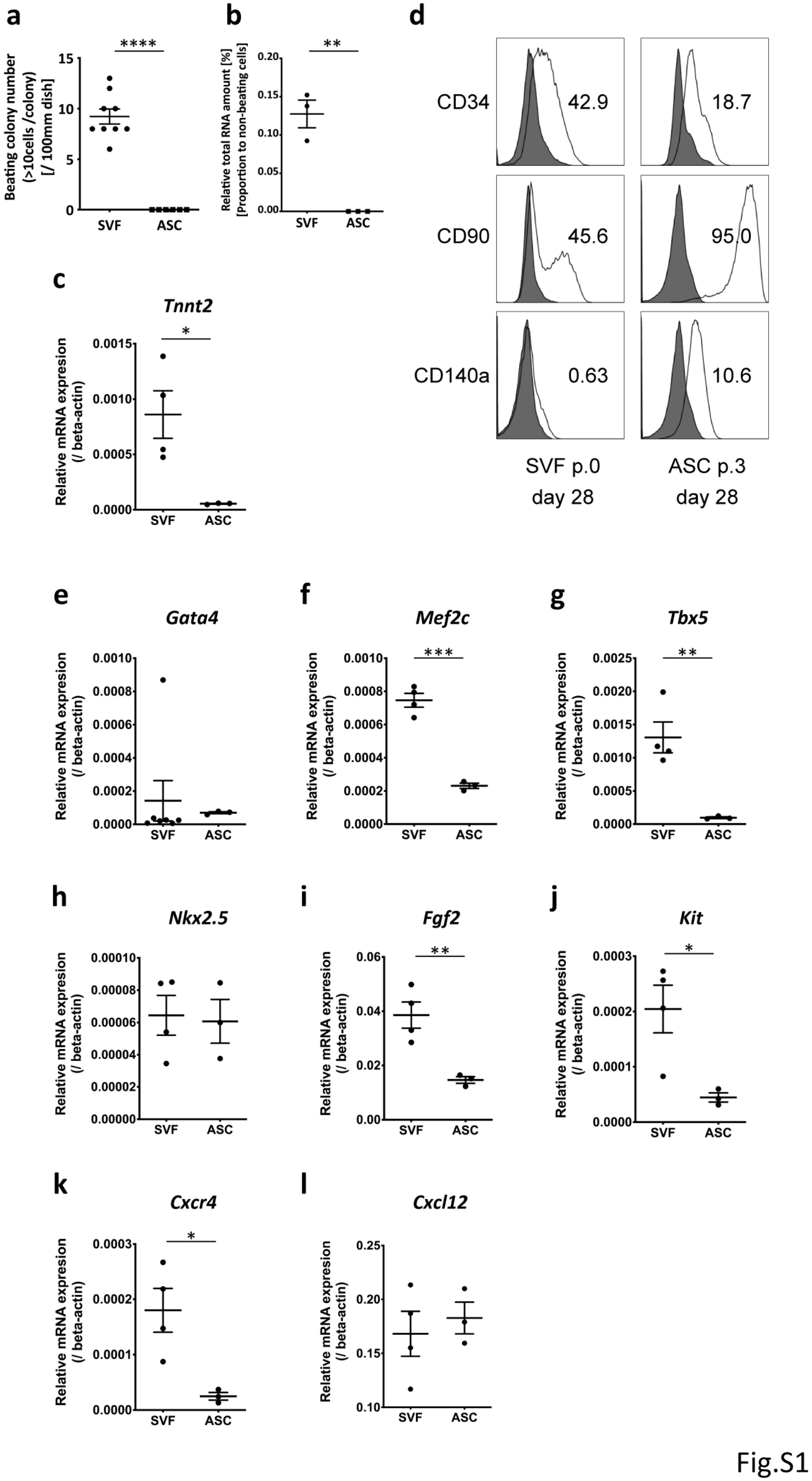
a
Beating colony number (>10cells /colony) [/ 100mm dish]
****
SVF
ASC
b
Relative total RNA amount [%] [Proportion to non-beating cells]
**
SVF
ASC
c
Tnnt2
*
d
CD34
42.9
18.7
CD90
45.6
95.0
CD140a
0.63
10.6
SVF p.0 day 28
ASC p.3 day 28
e
Gata4
f
Mef2c
***
g
Tbx5
**
h
Nkx2.5
i
Fgf2
**
j
Kit
*
k
Cxcr4
*
l
Cxcl12
Relative mRNA expresion (/ beta-actin)

Fig.S1

Figure S1. Transdifferentiation of SVF cells into spontaneously beating CMs in primary culture. (**a**, **b**) The number of colonies and relative total RNA level of beating cells on day 28 after plating SVF cells (passage 0) or ASCs (passage 3). SVF, stromal vascular fraction (passage 0), ASCs, adipose tissue-derived stem cells (passage 3) obtained by subculturing SVF cells three times. (**c**) mRNA level of Tnnt2 determined by qRT-PCR on day 28. (**d**) Flow cytometry of the stem cell surface markers CD34, CD90, and CD140a in SVF cells (passage 0) and ASCs (passage 3) on day 28, respectively. Gray peak, isotype control. (**e–l**) mRNA levels of key genes in SVF cells and ASCs on day 28 determined by qRT-PCR. * $p < 0.05$; ** $p < 0.01$; *** $p < 0.001$; **** $p < 0.0001$ by *t*-test.

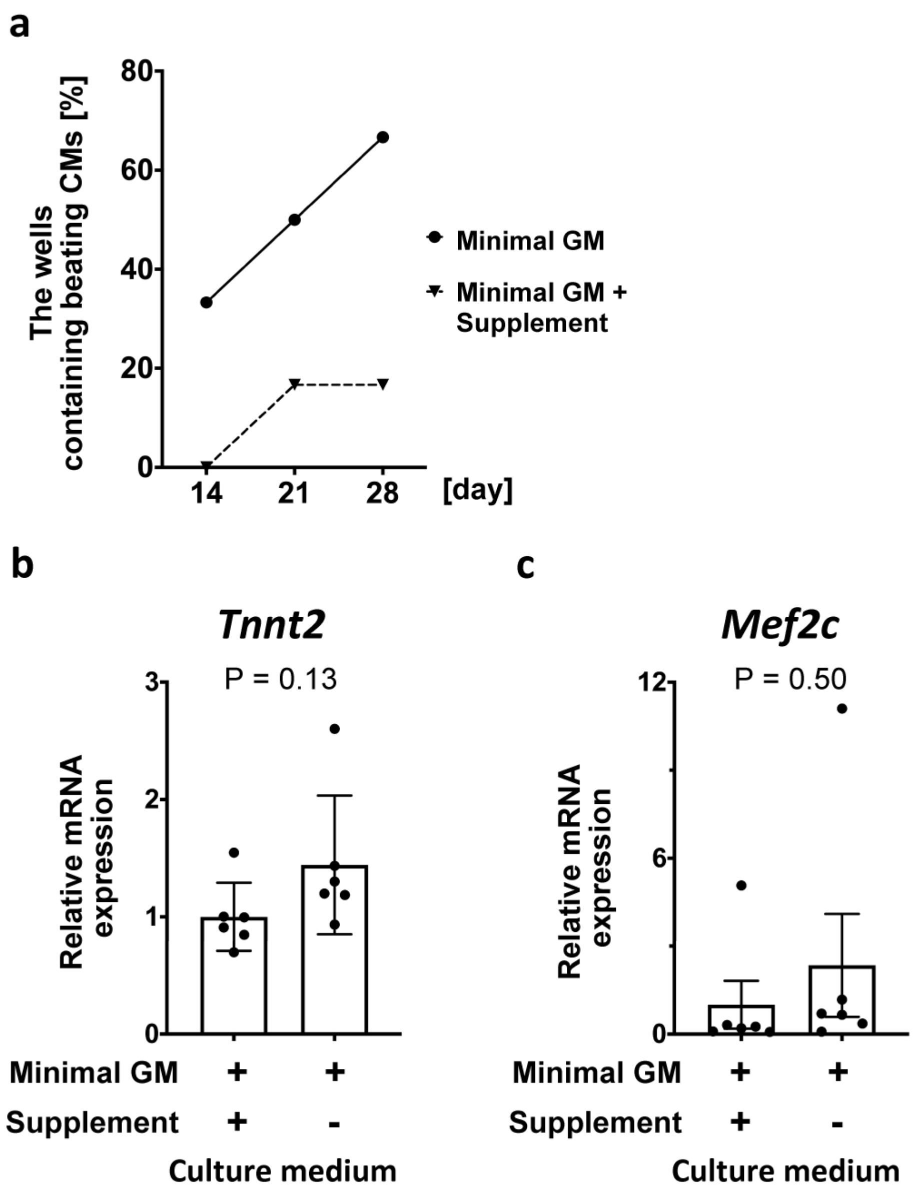
a
80
60
40
20
0
The wells containing beating CMs [%]
14
21
28
[day]
Minimal GM
Minimal GM + Supplement
b
Tnnt2
P = 0.13
3
2
1
0
Relative mRNA expression
Minimal GM + +
Supplement + -
Culture medium
c
Mef2c
P = 0.50
12
6
0
Relative mRNA expression
Minimal GM + +
Supplement + -
Culture medium

Fig.S2

Figure S2. Efficacy of the generation of beating SVFs in different growth media. (**a**) Frequency of wells with growth of beating SVF clusters in our minimal growth medium (GM) versus the complete GM (minimal GM plus supplements including growth factors and cytokines; see Methods) developed by Planat-Benard *et al*. (2004), on days 14, 21, and 28 of SVF cultivation. (b, c) The relative gene expression of Tnnt2 (b) and Mef2c (c) in all SVF cells on day 28 of cultivation using minimal versus complete GM. The *p*-values were determined by *t*-test.

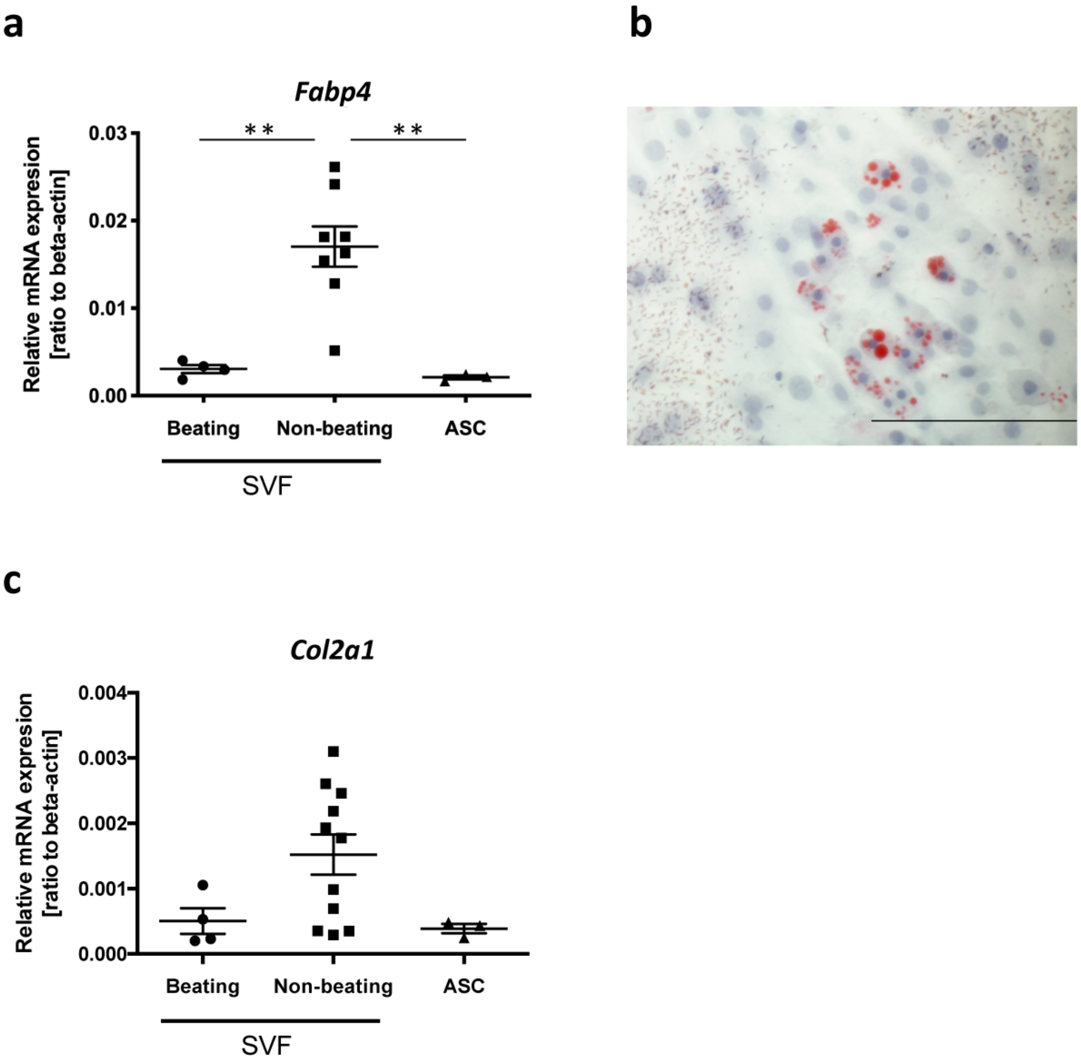
a
Fabp4
Relative mRNA expresion [ratio to beta-actin]
0.03
0.02
0.01
0.00
**
**
Beating
Non-beating
ASC
SVF
b
c
Col2a1
Relative mRNA expresion [ratio to beta-actin]
0.004
0.003
0.002
0.001
0.000
Beating
Non-beating
ASC
SVF

Fig.S3

Figure S3. Characteristics of SVF and ASCs that transdifferentiate into adipocyte and cartilage lineages. (**a**) mRNA level of Fabp4 in beating and non-beating cells in SVF and ASC on day 28. (**b**) Oil Red O staining of non-beating cells in SVF on day 28. (**c**) mRNA level of Col2a1 in beating and non-beating cells in SVF and ASC on day 28. ** $p < 0.01$ by ANOVA. Scale bar, 200 μm.

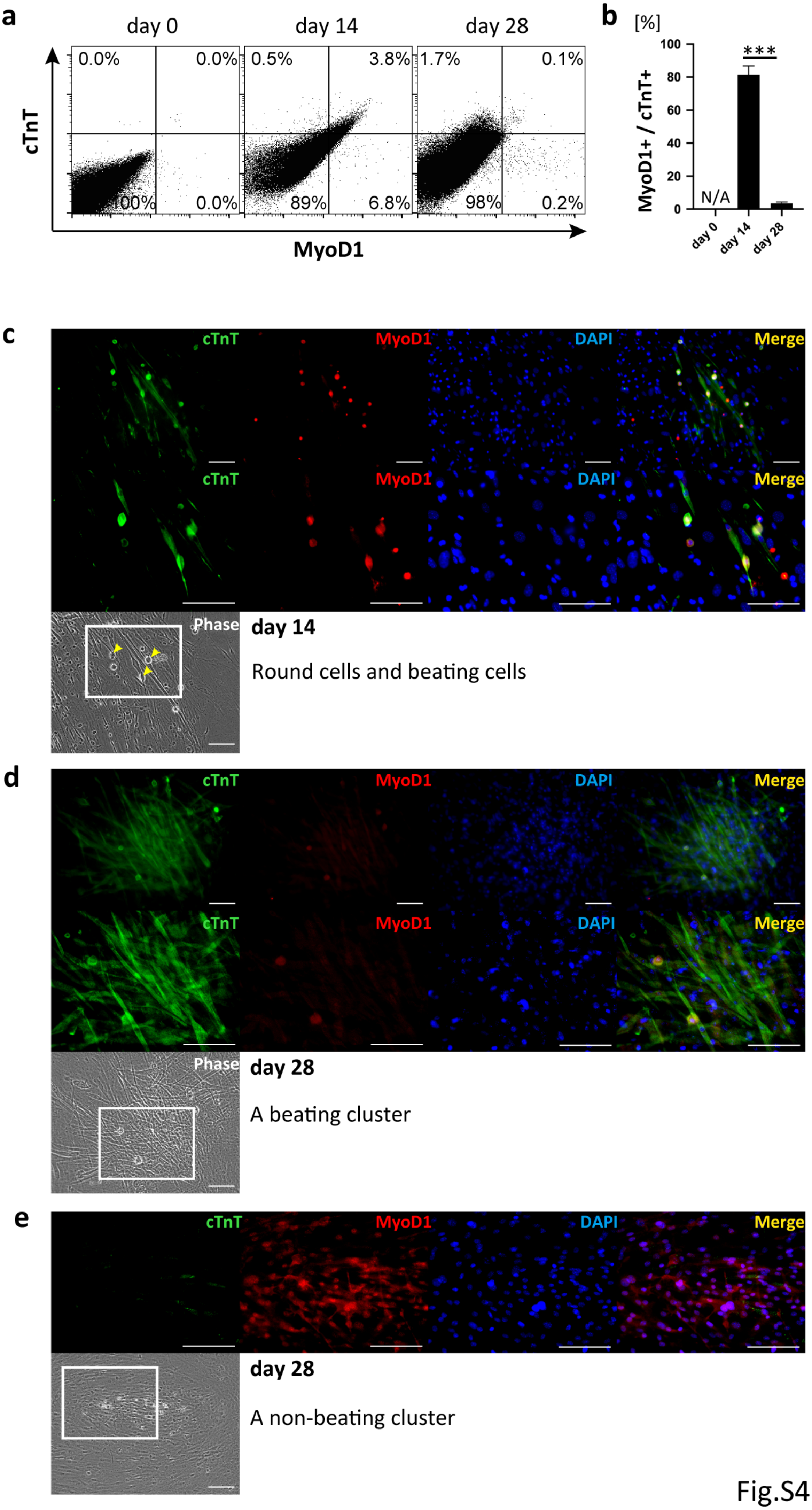

a
day 0
day 14
day 28
cTnT
MyoD1
0.0%
0.0%
100%
0.0%
0.5%
3.8%
89%
6.8%
1.7%
0.1%
98%
0.2%
b
[%]
MyoD1+ / cTnT+
***
N/A
day 0
day 14
day 28
c
cTnT
MyoD1
DAPI
Merge
Phase
day 14
Round cells and beating cells
d
cTnT
MyoD1
DAPI
Merge
Phase
day 28
A beating cluster
e
cTnT
MyoD1
DAPI
Merge
day 28
A non-beating cluster

Fig.S4

Figure S4. Gene and protein expression of MyoD1 and cardiac troponin T in SVF cells during primary culture. (**a**) Flow cytometric analysis of SVF cells before (day 0) and after cultivating for 14 and 28 days. The cells were stained with APC-conjugated MyoD1 and FITC-conjugated cardiac troponin T (cTnT) antibodies. The number in each quadrant indicates the percentage of cells. (**b**) The ratios of the number of MyoD1-positive cells to the number of cTnT-positive cells on days 14 and 28. ** $p < 0.001$ by *t*-test. (**c**) Representative immunofluorescence images of round cells and beating cells stained for cardiac troponin T (cTnT) and MyoD1 on day 14 after cultivating SVF cells. The arrowhead indicates a round cell. (**d, e**) Representative immunofluorescence images of beating (**d**) and non-beating (**e**) clusters stained for cTnT and MyoD1 on day 28 after cultivating SVF cells. White square, area of interest (shown above at a higher magnification). Scale bar, 100 μm.

Table S1. TaqMan assay ID of genes used in the TanMan qPCR

Gene	TaqMan assay ID
Tnnt2	Mm01290256_m1
Myh6	Mm00440359_m1
Actc1	Mm01333821_m1
Myl2	Mm00440384_m1
Myl7	Mm00491655_m1
Hcn4	Mm01176086_m1
Hcn2	Mm00468538_m1
Gja1	Mm00439105_m1
Nppb	Mm01255770_g1
Nppa	Mm01255748_g1
Gata4	Mm00484689_m1
Mef2c	Mm01340842_m1
Tbx5	Mm00803518_m1
Nkx2-5	Mm01309813_s1
Fgf2	Mm00433287_m1
Kit	Mm00445212_m1
Cxcr4	Mm01996749_s1
Cxcl12	Mm00445553_m1
Fabp4	Mm00445878_m1
Col2a1	Mm01309565_m1

Genes	Catalog number
mACTB (VIC^{TM})	4352341E

Table S2. The accession IDs in NCBI's Gene Expression Omnibus of which data were used.

**Platform**	GPL6246<br>[MoGene-1_0-st] Affymetrix Mouse Gene 1.0 ST Array [transcript (gene) version]
**Series**	**Sample**
GSE14414	GSM360098: heart_WT_rep1
GSE14414	GSM360099: heart_WT_rep2
GSE14414	GSM360100: heart_WT_rep3
GSE14414	GSM360108: embryo cardiomyocyte_rep1
GSE14414	GSM360109: embryo cardiomyocyte_rep2
GSE14414	GSM360110: embryo cardiomyocyte_rep3
GSE22292	GSM554758: neonatal cardiomyocyte_rep1
GSE22292	GSM554759: neonatal cardiomyocyte_rep2
GSE22292	GSM554760: neonatal cardiomyocyte_rep3
GSE24940	GSM613012: muscle1_rep1
GSE24940	GSM613013: muscle1_rep2
GSE24940	GSM613014: muscle1_rep3

Titles of the Supplementary videos

<u>All of the videos were original and the speed was not altered.</u>

Supplementary video S1. Representative beating SVF on day 12 of a primary culture of Ad-SVF; related to Fig. 1a.

Supplementary video S2. Representative beating SVF on day 14 of a primary culture of Ad-SVF; related to Fig. 1b.

Supplementary video S3. Representative beating SVF on day 20 of a primary culture of Ad-SVF (low magnification); related to Fig. 1c.

Supplementary video S4. Representative beating SVF on day 20 of a primary culture of Ad-SVF (high magnification); related to Fig. 1c.

Supplementary video S5. Representative beating SVF on day 28 of a primary culture of Ad-SVF (low magnification); related to Fig. 1d.

Supplementary video S6. Representative beating SVF on day 28 of a primary culture of Ad-SVF (High magnification); related to Fig. 1d.

Supplementary video S7. Phase-contrast movie of a cluster of SVF beating cells on day 14; related to Fig. 1e.

Supplementary video S8. Intracellular calcium oscillation in round cells and elongated-tube-like cells on day 14 that corresponds to video 7; related to Fig. 1f.

Supplementary video S9. Intracellular calcium oscillation in the cluster of beating SVF cells on day 28; related to Fig. 1h and 1i.

Supplementary video S10. Phase-contrast movie of beating SVF cell co-expressing cardiac troponin T with Mef2c; related to Fig. 6c.
